# Supplementary material for: Optical tools for visualizing and controlling human GLP-1 receptor activation with high spatiotemporal resolution
Source: eLife. 2023 Jun 2;12:RP86628. doi: 10.7554/eLife.86628 (PMC10262203; doi:10.7554/eLife.86628)
Supplement: Source data 1. [file elife-86628-data1.pdf]

Synthesis and Characterization of Building Blocks and Peptides

1 Materials and Equipment

Unless otherwise noted, all reactions were performed under an atmosphere of inert gas (N_2). The reactions were carried out in oven-dried glassware using dry solvents, unless otherwise noted. Room temperature is defined as a range between 20–25 °C.

1.1 Chemicals and Solvents

All chemicals and solvents were used as supplied, unless otherwise stated. Fmoc- and side chain-protected L-amino acids (Fmoc-Ala-OH, Fmoc-Arg(Pbf)-OH, Fmoc-Asn(Trt)-OH, Fmoc-Asp(*t*Bu)-OH, Fmoc-Cys(Trt)-OH, Fmoc-Gln(Trt)-OH, Fmoc-Glu(*t*Bu)-OH, Fmoc-Gly-OH, Fmoc-His(Trt)-OH, Fmoc-Ile-OH, Fmoc-Leu-OH, Fmoc-Lys(Boc)-OH, Fmoc-Met-OH, Fmoc-Phe-OH, Fmoc-Pro-OH, Fmoc-Ser(*t*Bu)-OH, Fmoc-Thr(*t*Bu)-OH, Fmoc-Trp(Boc)-OH, Fmoc-Tyr(*t*Bu)-OH, Fmoc-Val-OH) were purchased from Bachem AG or the Novabiochem-line from Sigma-Aldrich Chemie GmbH (Pbf = 2,2,4,6,7-pentamethyldihydrobenzofuran-5-sulfonyl, Trt = trityl, *t*Bu = *tert*-butyl, Boc = *tert*-butoxycarbonyl). Piperidine (99%) was purchased from Chemie Brunschwig AG. *O*-(7-Azabenzotriazol-1-yl)-*N,N,N',N'*-tetramethyluronium hexafluorophosphate (HATU) and (7-azabenzotriazol-1-yloxy)tripyrrolidinophosphonium hexafluorophosphate (PyAOP) were purchased from Bachem AG and Advanced ChemTech CreoSalus®, respectively. *N,N*-Diisopropylethylamine (*i*Pr_2NEt, DIPEA, 99.5%), trifluoroacetic acid (TFA, for HPLC, ≥99.0%), triisopropylsilane (TIPS, 98%), 3,6-dioxa-1,8-octane-dithiol (DODT, 95%), α-methyl-5-methoxy-2-nitro-4-(2-propyn-1-yloxy)benzyl alcohol, and acetonitrile (MeCN, HPLC gradient grade, ≥99.9%) were purchased from Sigma-Aldrich Chemie GmbH. *N,N'*-disuccinimidyl carbonate was purchased from the Novabiochem-line from Sigma-Aldrich Chemie GmbH. *N,N*-Dimethylformamide (DMF) was purchased from the Supelco-line from VWR International GmbH. Dichloromethane (DCM, ≥99.8%) was purchased from Fischer Scientific Inc. Diethyl ether was purchased from Honeywell Riedel-de Haën. Dry MeCN (99.9%, AcroSeal®, stored over molecular sieves) was purchased from Acros Organics. Hexanes (technical grade) and EtOAc (technical grade) used for flash column chromatography were purchased from Thommen-Furler AG and distilled under reduced pressure prior to use. NovaPEG Rink Amide resins (0.41 mmol/g and 0.20 mmol/g loading) were purchased from the Novabiochem-line from Sigma-Aldrich Chemie GmbH. Et_3N (anhydrous) was purchased from Fluorochem Ltd. AldraAmine trapping packets (volume 1000–4000 mL) were purchased from Sigma-Aldrich Chemie GmbH.

1.2 Chromatography

Analytical thin-layer chromatography (TLC): Merck TLC plates (silica gel 60) on glass with the indicated solvent system. TLC spots were visualized by UV light (254 nm) and by stains of $KMnO_4$ or Ninhydrin. Flash column chromatography was performed using Merck silica gel 60 (40−63 µm particle size) with the indicated solvent system.

1.3 UV-Vis Spectroscopy

UV-Vis spectra were acquired using a Multiskan SkyHigh Microplate Spectrophotometer (ThermoFisher Scientific Inc.) in the range of 200–450 nm with 1 nm steps and quartz cuvettes (10 mm pathlength, Hellma Schweiz AG).

1.4 NMR Spectroscopy

Nuclear magnetic resonance (NMR) spectra were recorded in deuterated chloroform ($CDCl_3$) or D_2O in 5 mm tubes on a Bruker AV2-401 (400 MHz) spectrometer equipped with a BOSS-I shim system, a digital lock control unit, an AMOS Control System, a DQD unit, a BVT3200 with a BCU05 cooling unit, GRASP Level II for gradient spectroscopy. Chemical shifts (δ scale) are expressed in parts per million (ppm) and are calibrated using residual protic solvent as an internal reference ($CHCl_3$: δ = 7.26 ppm). Data for ^1H NMR (400 MHz) spectra are reported as follows: chemical shift (δ ppm) (multiplicity, coupling constants (Hz), integration). Couplings are expressed as: s = singlet, d = doublet, t = triplet, q = quartet, m = multiplet or combinations thereof. ^{13}C NMR spectra were recorded at 126 MHz. Carbon chemical shifts (δ scale), expressed in parts per million (ppm), are referenced to the central carbon resonances of the solvents ($CHCl_3$: δ = 77.2 ppm).

1.5 Analytical LC-HR-ESI-MS (LCMS)

Liquid chromatography high resolution electrospray ionization mass spectrometry (LCMS): Acquity UPLC (Waters, Milford, USA) connected to an Acquity eλ diode array detector and a Synapt G2HR-ESI-QTOF-MS (Waters, Milford, USA); injection of 10 µL sample (c = *ca.* 10–100 µg/mL in the indicated solvent); Acquity BEH C8 HPLC column (1.7 µm particle size, 2.1 × 100 mm, Waters) kept at room temperature; elution at a flow rate of 0.3 mL/min with A: H_2O + 0.02% HCO_2H + 0.04% CF_3CO_2H and B: CH_3CN + 0.04% HCO_2H + 0.02% CF_3CO_2H, isocratic 5% B for 1 min; then 5–95% B over 9 min. UV-Vis spectra recorded in the range of 190–300 nm at 1.2 nm resolution and 20 points s^{-1}; ESI: positive ionization mode, capillary voltage 3.0 kV, sampling cone 40 V, extraction cone 4 V, N_2 cone gas 4 L/h, N_2 desolvation gas 800 L/min, source temperature 120 °C; mass analyzer in resolution mode: mass range 150–3000 m/z with a scan rate of 1 Hz; mass calibration to <2 ppm within 50–2500 m/z with a 5 mM aq. soln. of HCO_2Na, lock masses: m/z 195.0882 (caffeine, 0.70 ng/mL) and 556.2771 (Leucine-enkephalin, 2.0 ng/mL). All mass spectra are integrated across Rt = 4–6 min of the total ion count (TIC). The areas indicated in grey are excluded from integration, due to presence of the injection peak and solvent-mixing baseline perturbation. For purity of the final peptides, please refer to the UHPLC spectra. Deconvoluted masses from the raw m/z values are calculated using Mestrelab Research S.L.© MestReNova v. 14.1 Mnova MS Suite.

1.6 Analytical Ultra High-Performance Liquid Chromatography (UHPLC)

For determination of purity by UHPLC, the filtered peptide solution was diluted in 10–50% acetonitrile (MeCN) in water with 0.1% TFA (500 µL) to a final concentration of approximately 0.5 mg/mL. The samples were measured on Agilent 1290 Infinity II Series UHPLC with UV detection at 214 nm and analyzed using

Agilent OpenLab CDS and ChemStation software. Where specified, analytical UHPLC spectra were acquired wherein eluent A = MeCN/H_2O (5:95) with 0.1% TFA, and eluent B = MeCN/H_2O (95:5) with 0.1% TFA using either (i) an Agilent Zorbax 300SB-C18 Narrow-Bore column (2.1 mm × 150 mm, 5 μm particle size) kept at 40 °C, at a flow rate of 1.5 mL/min, with a gradient of 0% B for 3 min, followed by 0–100% B over 30 min (ca. 3% MeCN/min), then 0% B for a further 2 min (total method time was 35 min), or (ii) an Agilent Zorbax Eclipse Plus C18 Rapid Resolution HD column (2.1 × 50 mm, 1.8 μm particle size), with a gradient of 0% B for 1 min, followed by 0–100% B over 9 min (ca. 10% MeCN/min), then 0% B for a further 1 min (total method time was 11 min). Purities of the final peptides were calculated by integration of the Area Under the Curve (AUC) of desired product peak (detected at λ = 214 nm) as a percentage of the AUC of all peaks between the indicated timeframe.

2 General Procedures

2.1 Solid-Phase Automated Fast-flow Peptide Synthesis (AFPS)

Peptides were synthesized on an automated-flow system, which was built in the Hartrampf lab, based on the published AFPS system.[1] All peptides were prepared by AFPS on NovaPEG Rink Amide resin (0.41 mmol/g or 0.20 mmol/g, as specified) to afford C-terminally amidated peptides. All peptides were synthesized from the C- to N-terminus. Standard Fmoc/*t*Bu protected amino acids (0.40 M in DMF) were coupled to the solid support using HATU (0.38 M in DMF) or PyAOP (0.38 M in DMF) with DIPEA (neat, 3.0 mL/min) at an overall flow rate of 20 mL/min. Amino acids A, D, E, F, G, I, K, L, M, P, S, W, and Y were coupled using HATU. Amino acids N, Q, R, V, T, C, and H were coupled using PyAOP. All amino acids except C and H were preheated at 90 °C during the activation step with HATU or PyAOP, whereas C was preheated at 60 °C with PyAOP, and H was preactivated with PyAOP at room temperature. For amino acids D, E, F, G, I, K, L, M, P, S, W, and Y, each coupling was carried out for 19 sec at 20 mL/min. For amino acids A, C, H, N, Q, R, S, T, and V, couplings were carried out for 31 sec at 20 mL/min. Removal of the N^{α}-Fmoc group was achieved using 20% piperidine with 1% formic acid in DMF at a flow rate of 20 mL/min at 90 °C for 19 sec. Between each coupling and deprotection step, the resin was washed with DMF (32 mL) at 90 °C with a flow rate of 40 mL/min.

2.2 Cleavage of Peptidyl-Resins

After synthesis, the peptidyl-resin was washed with DCM (3 × 5 mL) and dried under reduced pressure. To the resin (amount as specified) was added cleavage solution (1.5–3.0 mL, TFA/TIPS/DODT/H_2O 94/1/2.5/2.5, *v*/*v*/*v*/*v*) and the reaction was allowed to proceed at room temperature for 2 h. Subsequently, the supernatant was collected by filtration and concentrated under a light stream of N_2. Thereafter, ice-cold diethyl ether (14 mL) was added to the concentrated supernatant, and the resulting precipitate collected as a pellet by centrifugation. The pellet was then suspended in a second portion of ice-cold diethyl ether (14 mL) and collected again by centrifugation. Residual ether was allowed to evaporate, and the peptide pellet was dissolved in 10–50% acetonitrile in water containing 0.1% TFA and lyophilized. Photocaged peptides were protected from light during all steps and handling.

2.3 Semi-Preparative Reverse-Phase High-Performance Liquid Chromatography (RP-HPLC)

All peptides were purified by semi-preparative RP-HPLC on a Shimadzu prominence HPLC system (Shimadzu Corp., Japan), using an Agilent Zorbax 300SB-C18 or 300SB-C8 Semi-Preparative column (9.4 × 250 mm, 5 μm particle size), wherein eluent A = H_2O with 0.1% TFA, and eluent B = MeCN with 0.1% TFA, and with UV absorbance detection at λ = 214 nm. Purifications were carried out at room temperature with a flow rate of 4.0 mL/min or 3.5 mL/min using linear gradients of eluents A and B as specified. Fractions (automatically collected) were then analyzed for purity by LCMS and UHPLC, and fractions containing the desired product (≥95% purity) were pooled and lyophilized.

2.4 Yield Calculations

For synthesis of peptides herein, a pre-loaded Novabiochem® NovaPEG Rink Amide resin (0.41 mmol/g or 0.20 mmol/g) was used. Theoretical yield was determined based on weight of the resin, resin loading, and the molecular weight of each purified peptide.

2.5 UV-Vis Spectroscopic Analysis of Peptides and PC Building Block

Individual UV-Vis spectra of Photo-GLP1, WT GLP-1, and α-methyl-5-methoxy-2-nitro-4-(2-propyn-1-yloxy)benzyl alcohol (PC Building Block) (80 μM in HBSS buffer) were acquired in the range of 200–450 nm with 1 nm steps. Absorbance values for each compound were plotted against wavelength.

2.6 Photocleavage and Characterization of Photocleaved Photo-GLP1

Photo-GLP1 (500 μL, 80 μM in HBSS buffer) was irradiated with a UVA-LED (λ = 370 nm, Kessil PR160L-370 nm) at maximal power settings (0.64 mW/mm^2) under air cooling. Samples (50 μL) were taken from the reaction at the following time-points: 20, 40, 120, and 300 seconds, and analyzed by LCMS. Another sample (50 μL) was taken at 300 seconds and combined with WT GLP1 (50 μL, 80 μM in HBSS buffer) and analyzed by LCMS to confirm identity of the photocleaved product.

3 Building Block Synthesis

1-(5-methoxy-2-nitro-4-(prop-2-yn-1-yloxy)phenyl)ethyl *N*-succinimidyl carbonate (SI1)[2]

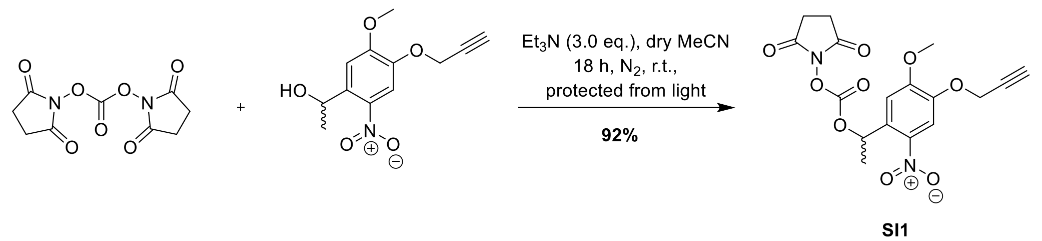

SI1

To a solution of dry acetonitrile (50 mL) was added α-methyl-5-methoxy-2-nitro-4-(2-propyn-1-yloxy)benzyl alcohol (0.10 g, 0.39 mmol, 1.0 eq.), *N,N'*-disuccinimidyl carbonate (0.23 g, 0.90 mmol, 2.3 eq.), and Et_3N (0.16 mL, 1.2 mmol, 3.0 eq.). The reaction was protected from light stirred under N_2 atmosphere at r.t. for 18 h. The reaction mixture was then concentrated under reduced pressure, and the product was isolated by flash column (3:1 followed by 2:1 hexanes:EtOAc) to yield the *title compound* **SI1** as an off-white solid (0.14 g, 92%). **Rf** 0.31 (1:1 hexanes:EtOAc). **IR** (film, $CHCl_3$) 3282.9, 1812.7, 1788.7, 1739.9, 1583.6, 1521.9, 1456.9, 1376.2, 1336.9, 1278.3, 1207.7, 1077.7, 1045.8, 1014.8. **HRMS** (ESI) (m/z): $[M+Na]^+$ calcd. for $C_{17}H_{16}N_2NaO_9$, 415.0748; found 415.0755. **^{1}H NMR** (400 MHz, $CDCl_3$) δ 7.83 (s, 1H), 7.11 (s, 1H), 6.52 (q, *J* = 6.4 Hz, 1H), 4.83 (dd, *J* = 2.5, 1.4 Hz, 2H), 4.06 (s, 3H), 2.80 (s, 4H), 2.59 (t, *J* = 2.4 Hz, 1H), 1.77 (d, *J* = 6.4 Hz, 3H). **^{13}C NMR** (126 MHz, $CDCl_3$) δ 168.58, 154.90, 150.73, 146.18, 139.17, 132.36, 110.62, 107.73, 57.15, 56.77, 25.56, 22.10.

Spectroscopic data was in good agreement with the literature.[3]

^{1}H NMR (400 MHz, CDCl$_3$) 1-(5-methoxy-2-nitro-4-(prop-2-yn-1-yloxy)phenyl)ethyl *N*-succinimidyl carbonate (**SI1**)

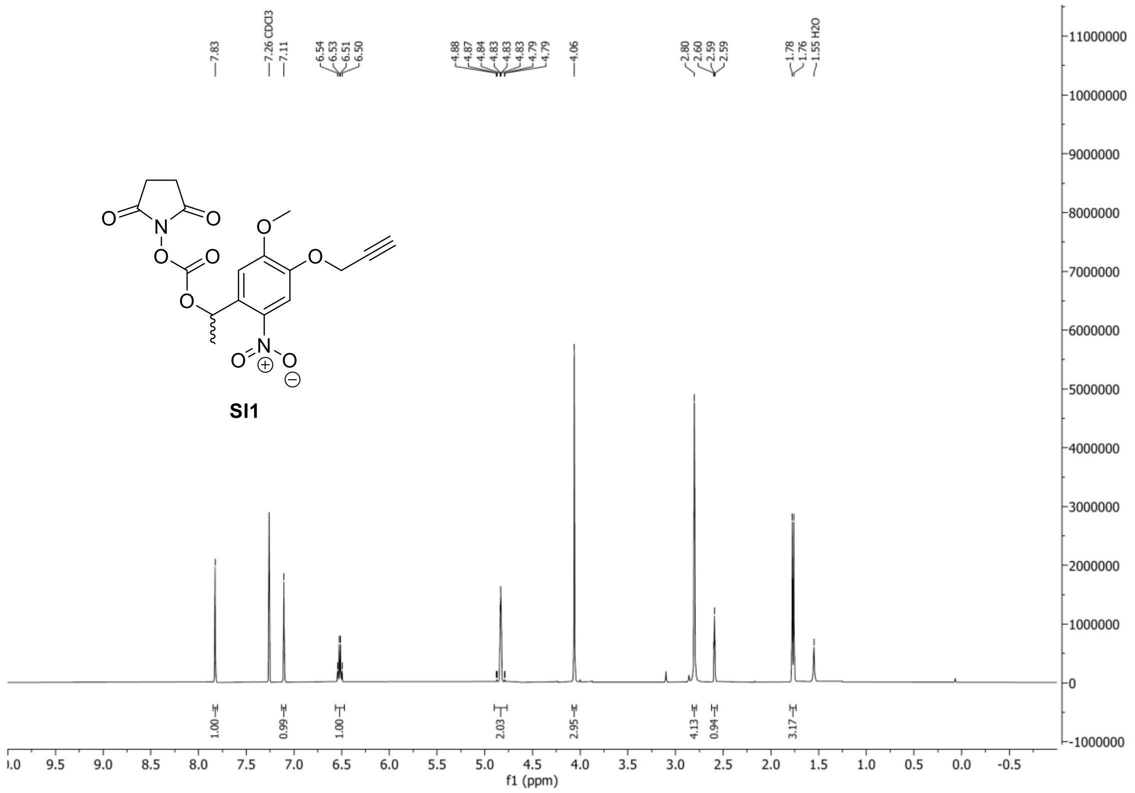

^{13}C NMR (126 MHz, CDCl$_3$) 1-(5-methoxy-2-nitro-4-(prop-2-yn-1-yloxy)phenyl)ethyl *N*-succinimidyl carbonate (**SI1**)

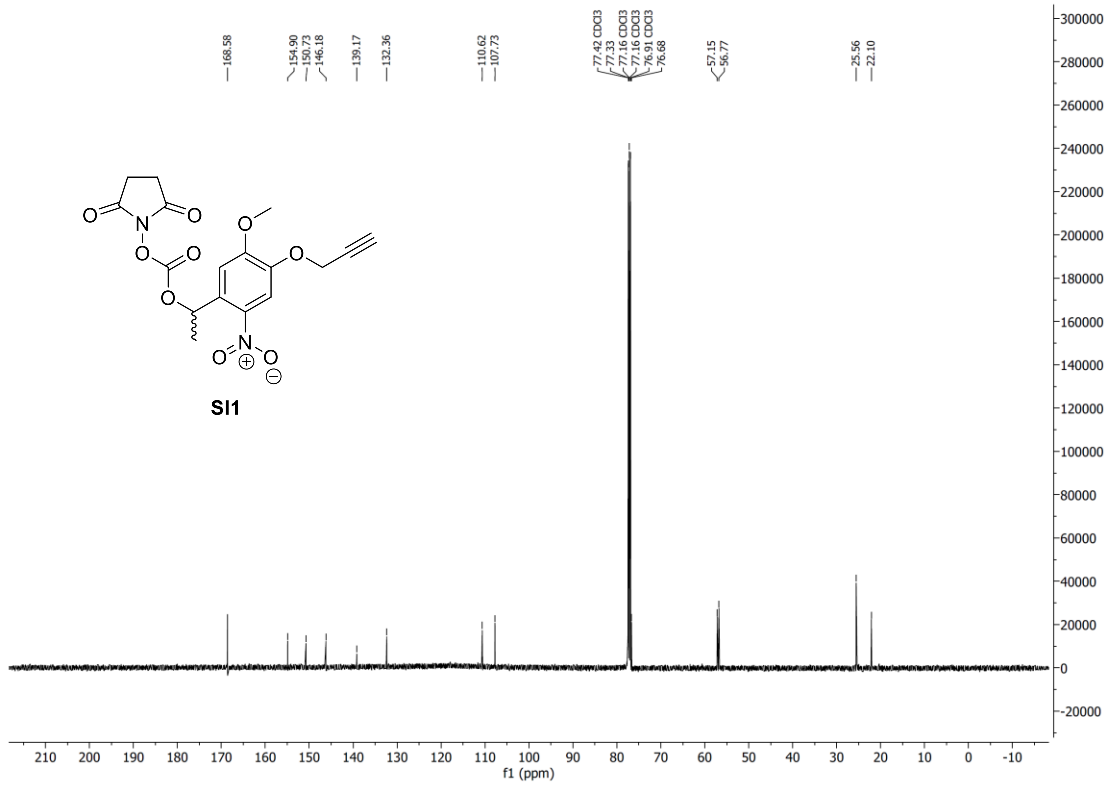

4 Peptide Synthesis

Synthesis of GLP1[6-30]

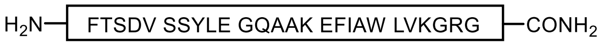

GLP1[6-30]
after mini-cleavage

The peptide GLP1[6-30] was prepared via AFPS using Novabiochem® NovaPEG Rink Amide resin (0.41 mmol/g, 0.15 g, 62 μmol) using methods outlined in **General Procedure 2.1**. Total synthesis time to afford resin-bound GLP-1 was approximately 1.5 h. Mini-cleavage of the peptidyl-resin (5.0 mg) was carried out using **General Procedure 2.2** to confirm presence of the crude peptide (67% purity by LCMS, monoisotopic mass calc. 2800.4548, found 2800.7377), **SI Figure 1**. The remaining peptidyl-resin was divided into four equal portions (approx. 16 μmol each) for the synthesis of alanine scan analogues of GLP-1; [H1A]GLP1, [E3A]GLP1, [G4A]GLP1 and [T5A]GLP1.

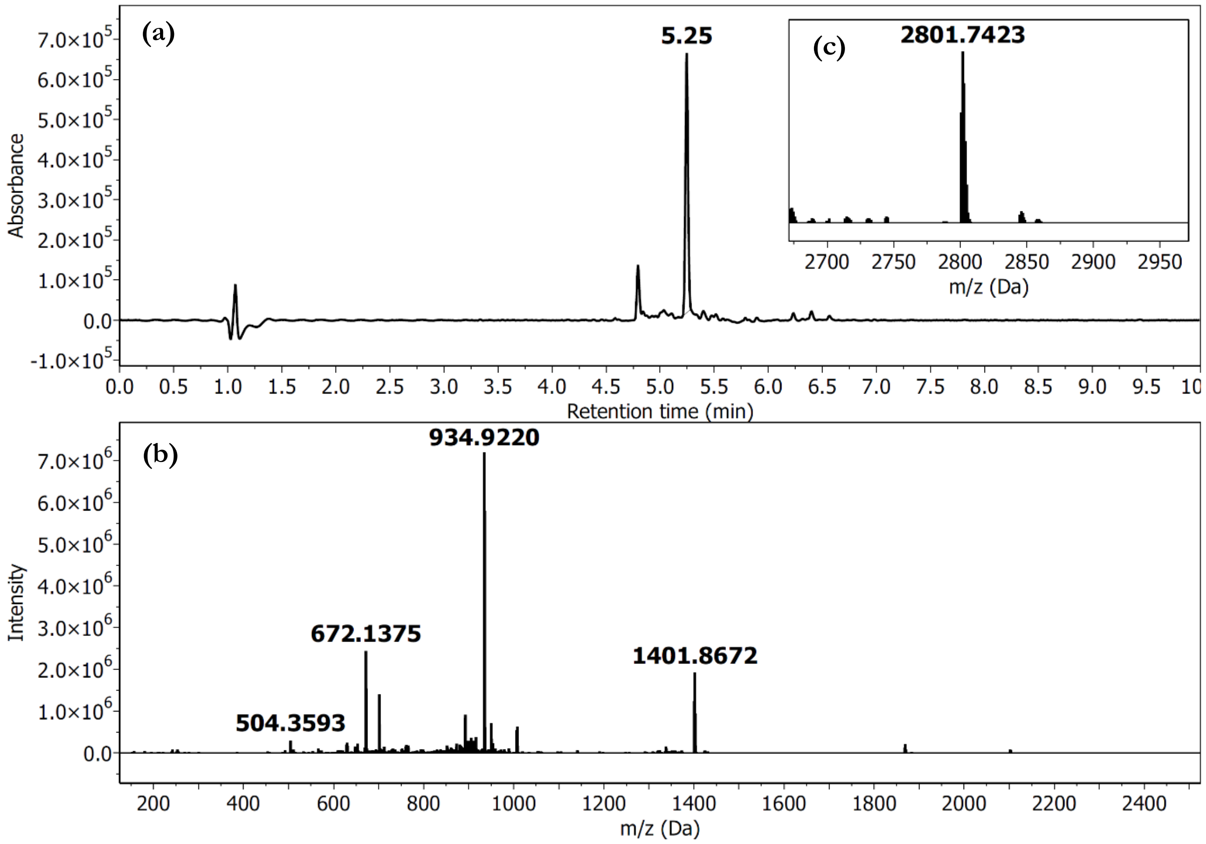

SI Figure 1. LCMS Profile of crude core peptide GLP1[6-30]. (**a**) Chromatogram (λ = 214 nm) of core peptide GLP1[6-30]; *R*t 5.246 min. (**b**) HRMS (ESI-TOF) spectrum. (**c**) Deconvoluted HRMS; monoisotopic mass calcd. for $C_{129}H_{197}N_{33}O_{37}$ 2800.4548, found 2800.7377.

Synthesis of [H1A]GLP1

H_2N— AAEGT FTSDV SSYLE GQAAK EFIAW LVKGRG —$CONH_2$

[H1A]GLP1

The peptide [H1A]GLP1 was prepared from resin-bound GLP1[6-30] (approx. 16 μmol) via AFPS using methods outlined in **General Procedure 2.1**. Total synthesis time to afford resin-bound [H1A]GLP1 (94 mg final resin weight) from GLP1[6-30] was approximately 20 min. Cleavage of the peptidyl-resin (50 mg) was carried out using **General Procedure 2.2** to afford the crude peptide (34 mg, 58% purity by UHPLC, monoisotopic mass calc. 3229.6408, found 3229.9870), **SI Figures 2** and **4**. The crude peptide (11 mg) was purified by semi-prep RP-HPLC using **General Procedure 2.3** with a gradient of 10–30% B over 20 min (*ca.* 1% B/min), followed by 30–50% B over 40 min (*ca.* 0.5% B/min) on an Agilent Zorbax 300SB-C18 column at room temperature. Fractions were analyzed by LCMS and UHPLC, and fractions containing the correct m/z and high purity (>95% purity by UHPLC) were combined and lyophilized to afford the *title compound* (2.6 mg, >95% purity by UHPLC, 25% overall yield, monoisotopic mass calc. 3229.6408, found 3229.9545), **SI Figures 3** and **5**.

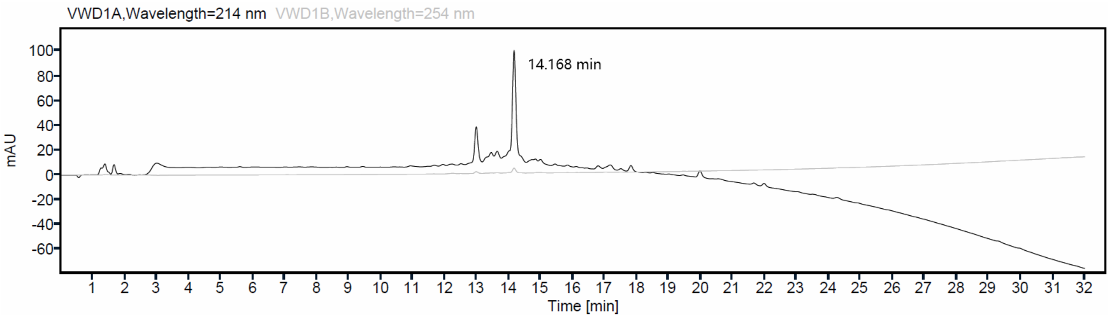

SI Figure 2. UHPLC profile of [H1A]GLP1 crude product. *R*t 14.168 min (Agilent Zorbax 300SB-C18 column, 5 μm, 2.1 × 150 mm, 5-95% MeCN over 30 min, *ca.* 3% MeCN/min), 58% purity based on Area Under Curve (AUC) at λ = 214 nm, accounting for all peaks between 4.0–29 min.

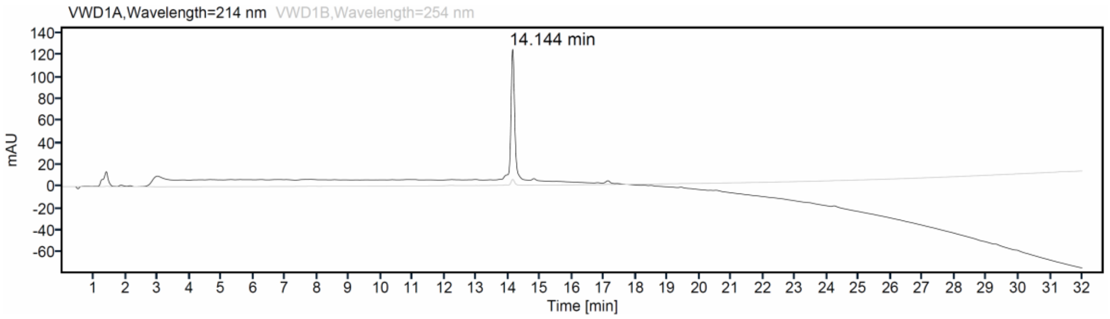

SI Figure 3. UHPLC profile of [H1A]GLP1 pure product. *R*t 14.144 min (Agilent Zorbax 300SB-C18 column, 5 μm, 2.1 × 150 mm, 5-95% MeCN over 30 min, *ca.* 3% MeCN/min), >95% purity based on Area Under Curve (AUC) at λ = 214 nm, accounting for all peaks between 4.0–29 min.

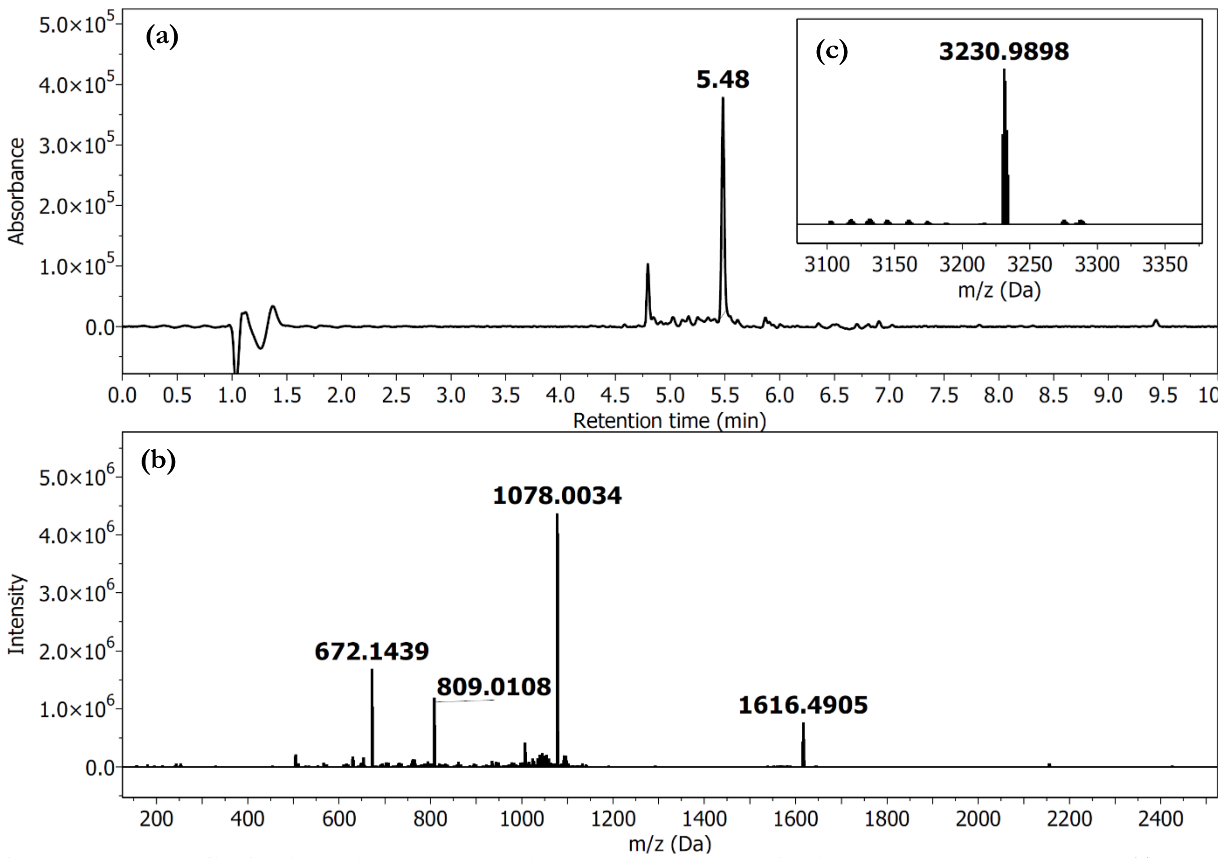

SI Figure 4. LCMS profile of crude peptide [H1A]GLP1. (**a**) Chromatogram (λ = 214 nm) of crude [H1A]GLP1; *R*t 5.482 min. (**b**) ESI-TOF. (**c**) Deconvoluted HRMS; monoisotopic mass calcd. for $C_{146}H_{224}N_{38}O_{45}$ 3229.6408, found 3229.9870.

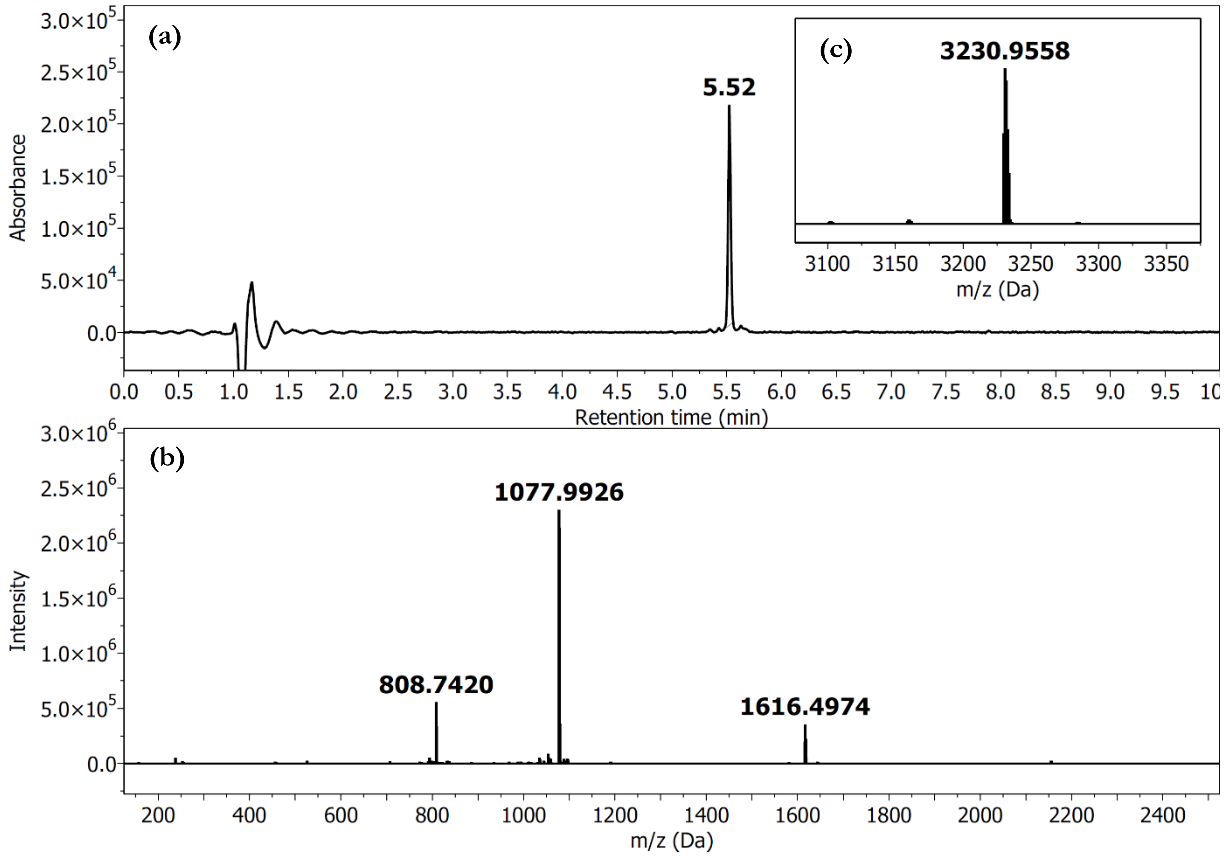

SI Figure 5. LCMS profile of pure peptide [H1A]GLP1. (**a**) Chromatogram (λ = 214 nm) of pure [H1A]GLP1; *R*t 5.523 min. (**b**) HRMS (ESI-TOF) spectrum. (**c**) Deconvoluted HRMS; monoisotopic mass calcd. for $C_{146}H_{224}N_{38}O_{45}$ 3229.6408, found 3229.9545.

Synthesis of [E3A]GLP1

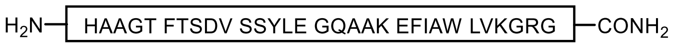

[E3A]GLP1

The peptide [E3A]GLP1 was prepared from resin-bound GLP1[6-30] (approx. 16 µmol) via AFPS using methods outlined in **General Procedure 2.1**. Total synthesis time to afford resin-bound [E3A]GLP1 (98 mg final resin weight) from GLP1[6-30] was approximately 20 min. Cleavage of the peptidyl-resin (52 mg) was carried out using **General Procedure 2.2** to afford the crude peptide (35 mg, 54% purity by UHPLC, monoisotopic mass calc. 3237.6571, found 3237.6608), **SI Figures 6** and **8**. The crude peptide (6.6 mg) was purified by semi-prep RP-HPLC using **General Procedure 2.3** with a gradient of 10–30% B over 20 min (*ca.* 1% B/min), followed by 30–50 % B over 40 min (*ca.* 0.5% B/min) on an Agilent Zorbax 300SB-C18 column at room temperature. Fractions were analyzed by LCMS and UHPLC, and fractions containing the correct m/z and high purity (>95% purity by UHPLC) were combined and lyophilized to afford the *title compound* (1.5 mg, >95% purity by UHPLC, 21% overall yield, monoisotopic mass calc. 3237.6571, found 3237.6512), **SI Figures 7** and **9**.

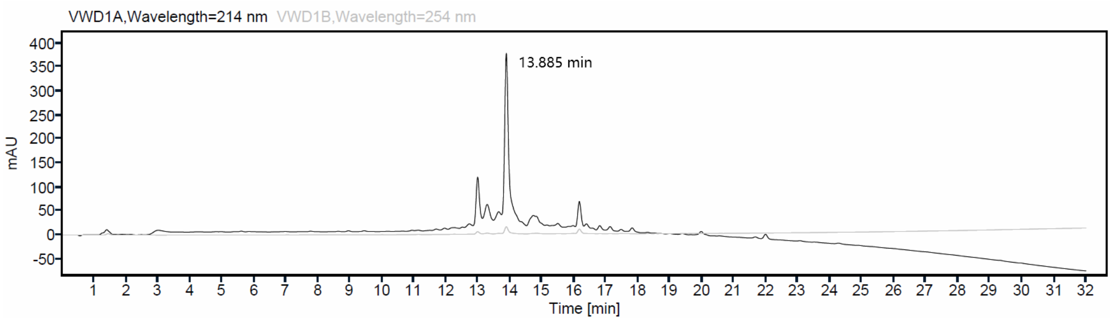

SI Figure 6. UHPLC profile of [E3A]GLP1 crude product. *Rt* 13.885 min (Agilent Zorbax 300SB-C18 column, 5 µm, 2.1 × 150 mm, 5-95% MeCN over 30 min, *ca.* 3% MeCN/min), 54% purity based on Area Under Curve (AUC) at λ = 214 nm, accounting for all peaks between 4.0–29 min.

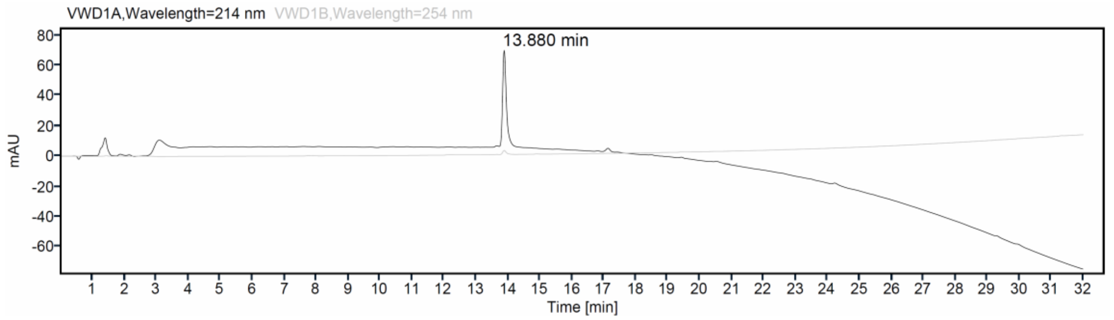

SI Figure 7. UHPLC profile of [E3A]GLP1 pure product. *Rt* 13.880 min (Agilent Zorbax 300SB-C18 column, 5 µm, 2.1 × 150 mm, 5-95% MeCN over 30 min, *ca.* 3% MeCN/min), >95% purity based on Area Under Curve (AUC) at λ = 214 nm, accounting for all peaks between 4.0–29 min.

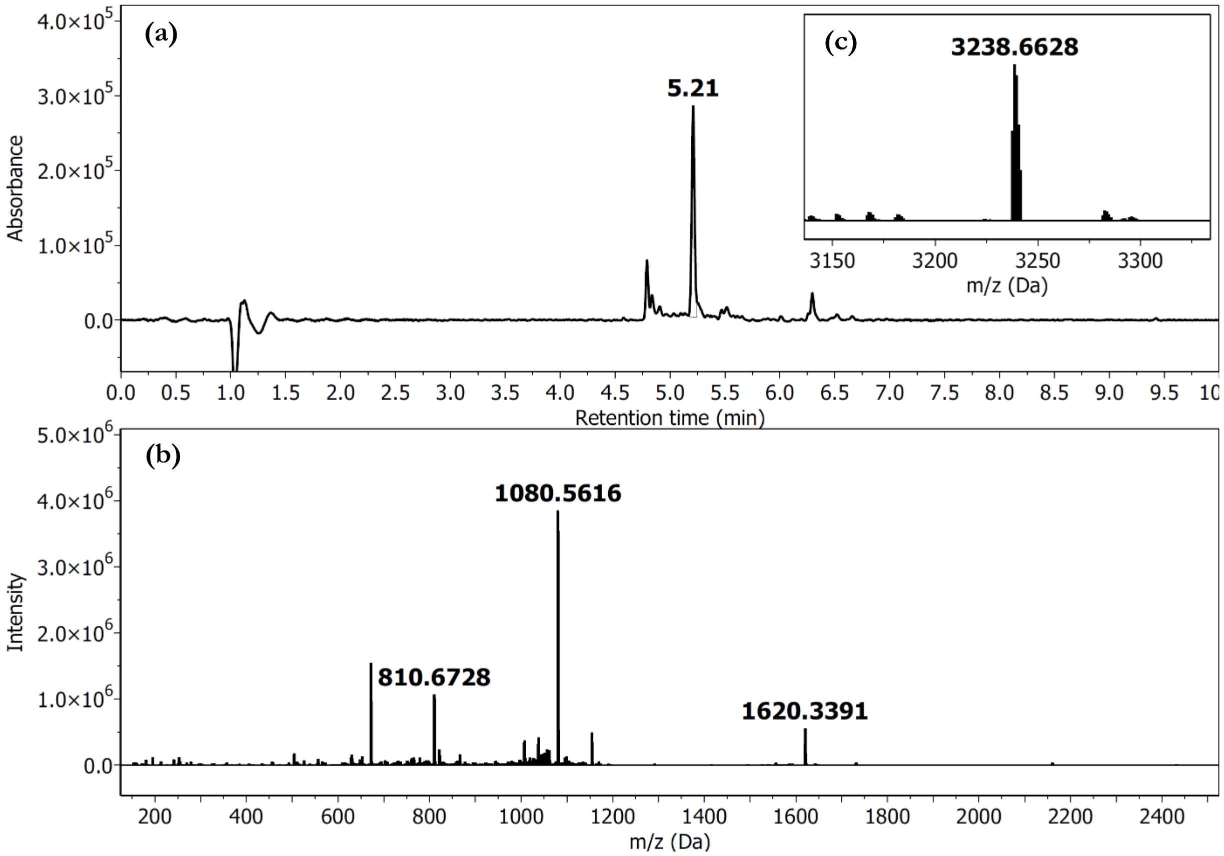

SI Figure 8. LCMS profile of crude peptide [E3A]GLP1. (**a**) Chromatogram (λ = 214 nm) of crude [E3A]GLP1; *R*t 5.210 min. (**b**) HRMS (ESI-TOF) spectrum. (**c**) Deconvoluted HRMS; monoisotopic mass calcd. for $C_{147}H_{224}N_{40}O_{43}$ 3237.6571, found 3237.6608.

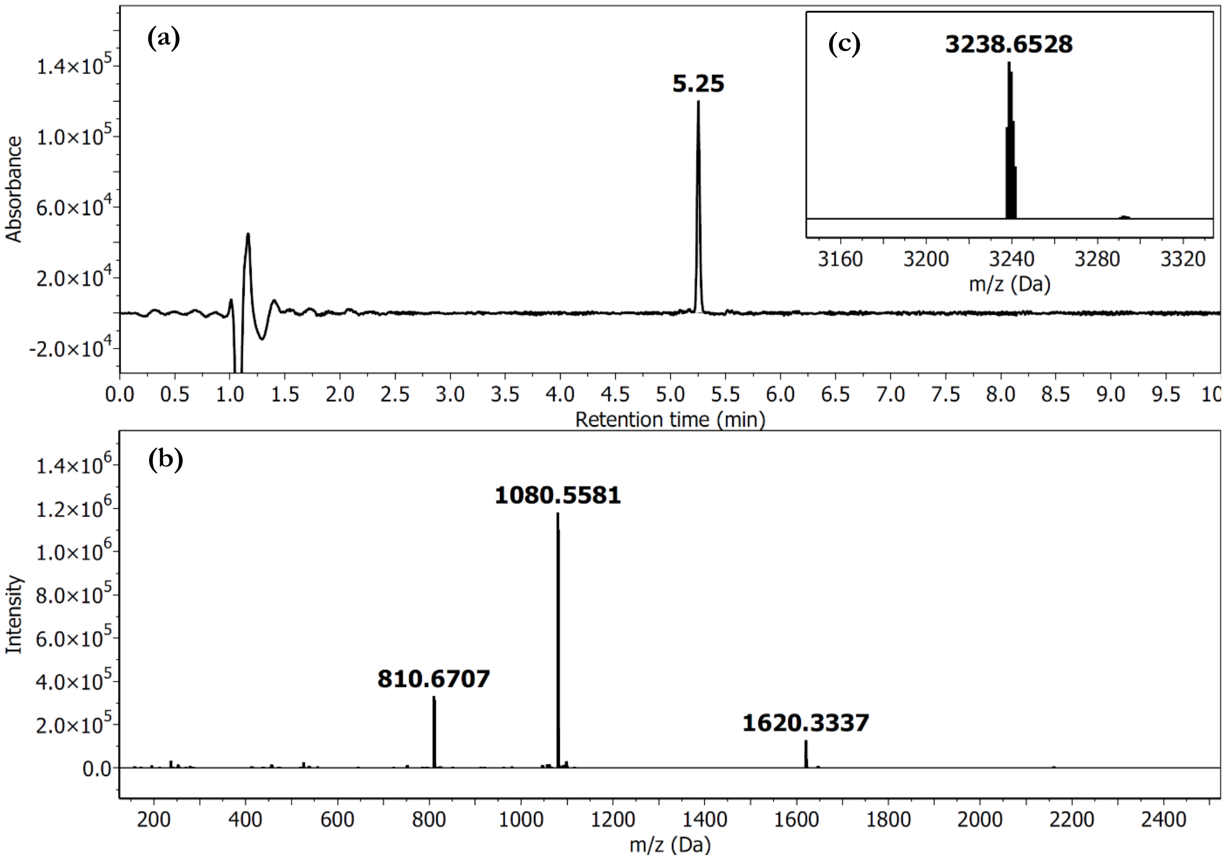

SI Figure 9. LCMS profile of pure peptide [E3A]GLP1. (**a**) Chromatogram (λ = 214 nm) of pure [E3A]GLP1; *R*t 5.253 min. (**b**) HRMS (ESI-TOF) spectrum. (**c**) Deconvoluted HRMS; monoisotopic mass calcd. for $C_{147}H_{224}N_{40}O_{43}$ 3237.6571, found 3237.6512.

Synthesis of [G4A]GLP1

H_2N— HAEAT FTSDV SSYLE GQAAK EFIAW LVKGRG —$CONH_2$

[G4A]GLP1

The peptide [G4A]GLP1 was prepared from resin-bound GLP1[6-30] (approx. 16 μmol) via AFPS using methods outlined in **General Procedure 2.1**. Total synthesis time to afford resin-bound [G4A]GLP1 (89 mg final resin weight) from GLP1[6-30] was approximately 20 min. Cleavage of the peptidyl-resin (47 mg) was carried out using **General Procedure 2.2** to afford the crude peptide (25 mg, 53% purity by UHPLC, monoisotopic mass calc. 3309.6782, found 3309.6778), **SI Figures 10** and **12**. The crude peptide (6.2 mg was purified by semi-prep RP-HPLC using **General Procedure 2.3** with a gradient of 10–30% B over 20 min (*ca.* 1% B/min), followed by 30–50% B over 40 min (*ca.* 0.5% B/min) on an Agilent Zorbax 300SB-C18 column at room temperature. Fractions were analyzed by LCMS and UHPLC, and fractions containing the correct m/z and high purity (>95% purity by UHPLC) were combined and lyophilized to afford the *title compound* (1.5 mg, 94% purity by UHPLC, 17% overall yield, monoisotopic mass calc. 3309.6782, found 3309.6696), **SI Figures 11** and **13**.

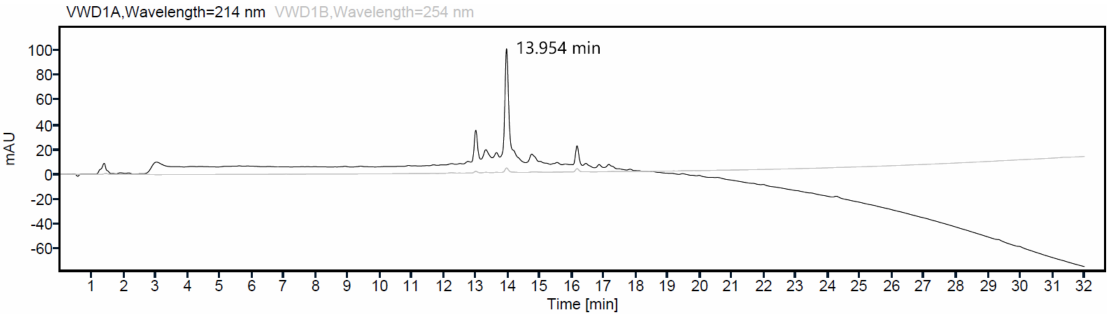

SI Figure 10. UHPLC profile of [G4A]GLP1 crude product. *R*t 13.954 min (Agilent Zorbax 300SB-C18 column, 5 μm, 2.1 × 150 mm, 5-95% MeCN over 30 min, *ca.* 3% MeCN/min), 53% purity based on Area Under Curve (AUC) at λ = 214 nm, accounting for all peaks between 4.0–29 min.

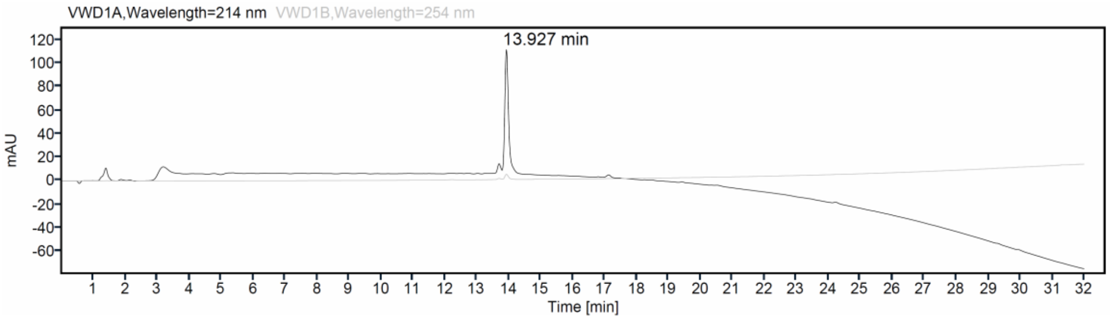

SI Figure 11. UHPLC profile of [G4A]GLP1 pure product. *R*t 13.927 min (Agilent Zorbax 300SB-C18 column, 5 μm, 2.1 × 150 mm, 5-95% MeCN over 30 min, *ca.* 3% MeCN/min), 94% purity based on Area Under Curve (AUC) at λ = 214 nm, accounting for all peaks between 4.0–29 min.

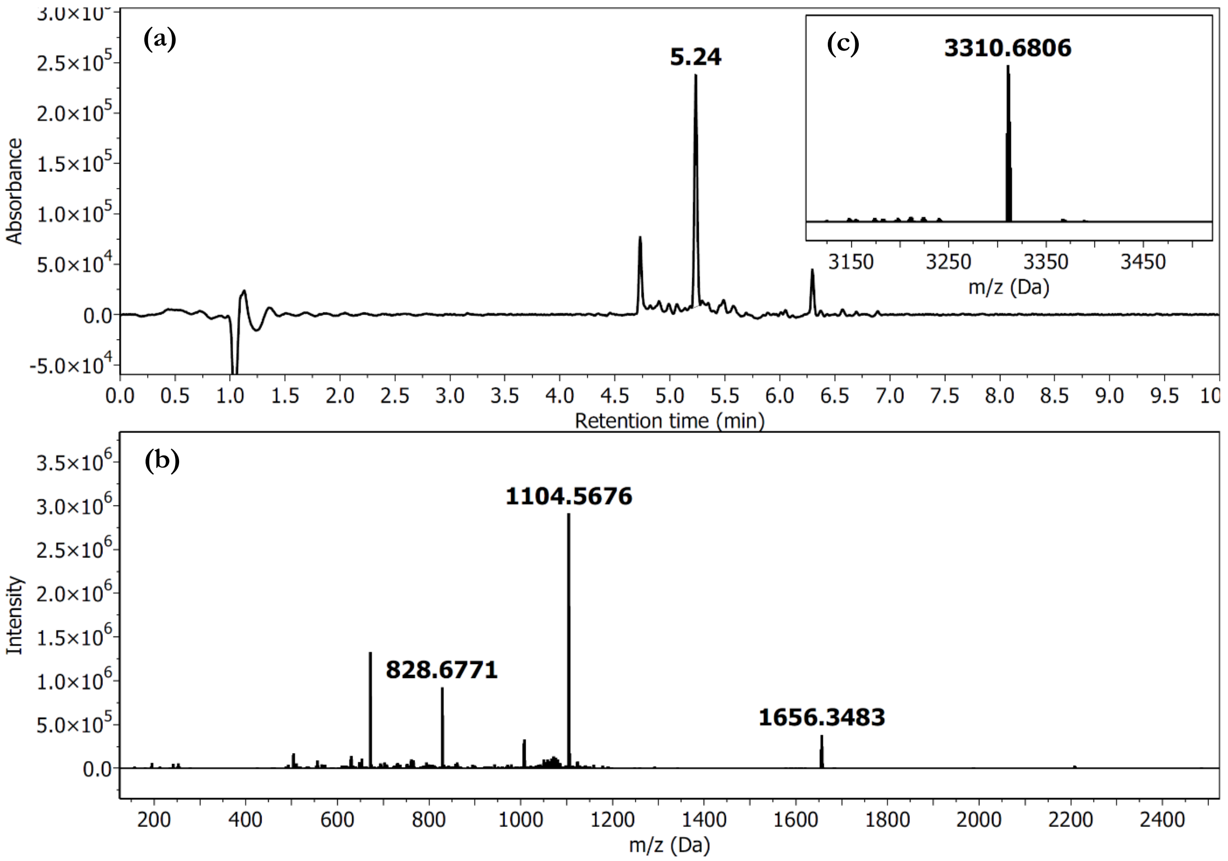

SI Figure 12. LCMS profile of crude peptide [G4A]GLP1. (**a**) Chromatogram (λ = 214 nm) of crude [G4A]GLP1; *R*t 5.235 min. (**b**) HRMS (ESI-TOF) spectrum. (**c**) Deconvoluted HRMS; monoisotopic mass (ESI+) calcd. for $C_{150}H_{228}N_{40}O_{45}$ 3309.6782, found 3309.6778.

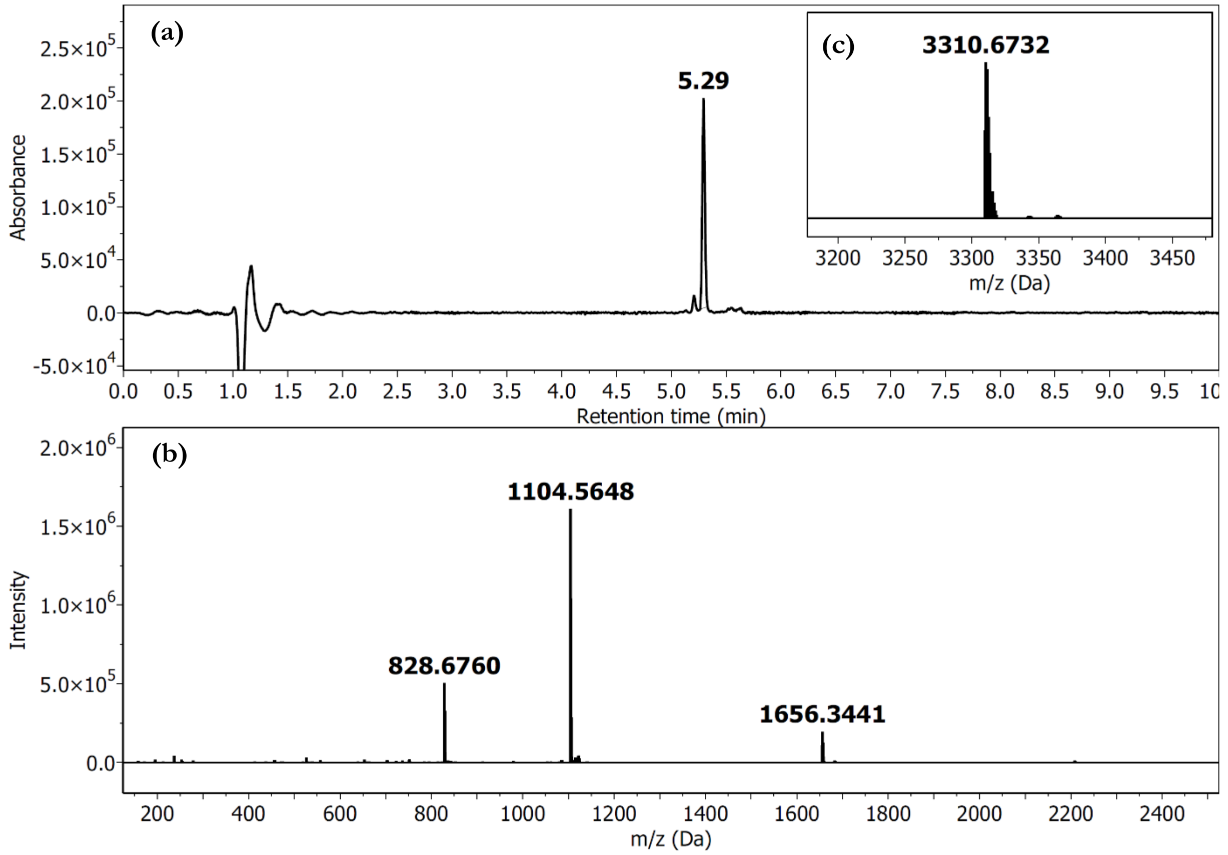

SI Figure 13. LCMS profile of pure peptide [G4A]GLP1. (**a**) Chromatogram (λ = 214 nm) of pure [G4A]GLP1; *R*t 5.294 min. (**b**) HRMS (ESI-TOF) spectrum. (**c**) Deconvoluted HRMS; monoisotopic mass (ESI+) calcd. for $C_{150}H_{228}N_{40}O_{45}$ 3309.6782, found 3309.6696.

Synthesis of [T5A]GLP1

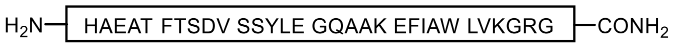

[G4A]GLP1

The peptide [T5A]GLP1 was prepared from resin-bound GLP1[6-30] (approx. 16 μmol) via AFPS using methods outlined in **General Procedure 2.1**. Total synthesis time to afford resin-bound [T5A]GLP1 (89 mg final resin weight) from GLP1[6-30] was approximately 20 min. Cleavage of the peptidyl-resin (49 mg) was carried out using **General Procedure 2.2** to afford the crude peptide (33 mg, 58% purity by UHPLC, monoisotopic mass calc. 3265.6520, found 3265.6516), **SI Figures 14** and **16**. The crude peptide (8.4 mg) was purified by semi-prep RP-HPLC using **General Procedure 2.3** with a gradient of 10–30% B over 20 min (*ca.* 1% B/min), followed by 30–50% B over 40 min (*ca.* 0.5% B/min) on an Agilent Zorbax 300SB-C18 column at room temperature. Fractions were analyzed by LCMS and UHPLC, and fractions containing the correct m/z and high purity (>95% purity by UHPLC) were combined and lyophilized to afford the *title compound* (0.84 mg, >95% purity by UHPLC, 9.3% overall yield, monoisotopic mass calc. 3265.6520, found 3265.6399), **SI Figures 15** and **17**.

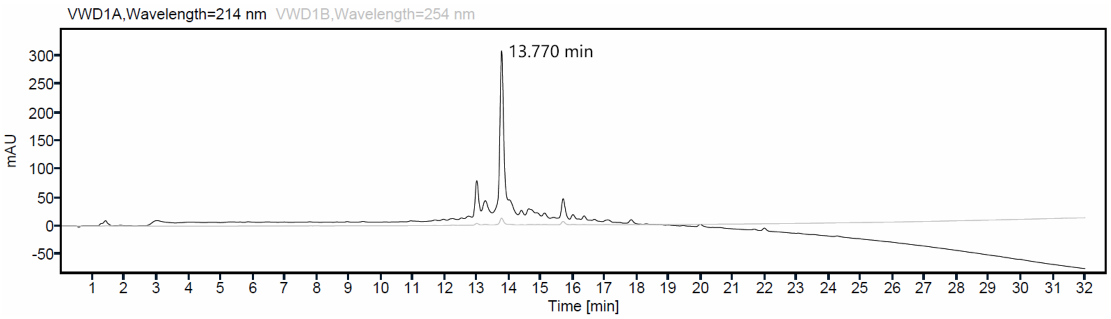

SI Figure 14. UHPLC profile of [T5A]GLP1 crude product. *R*t 13.770 min (Agilent Zorbax 300SB-C18 column, 5 μm, 2.1 × 150 mm, 5-95% MeCN over 30 min, *ca.* 3% MeCN/min), 58% purity based on Area Under Curve (AUC) at λ = 214 nm, accounting for all peaks between 4.0–29 min.

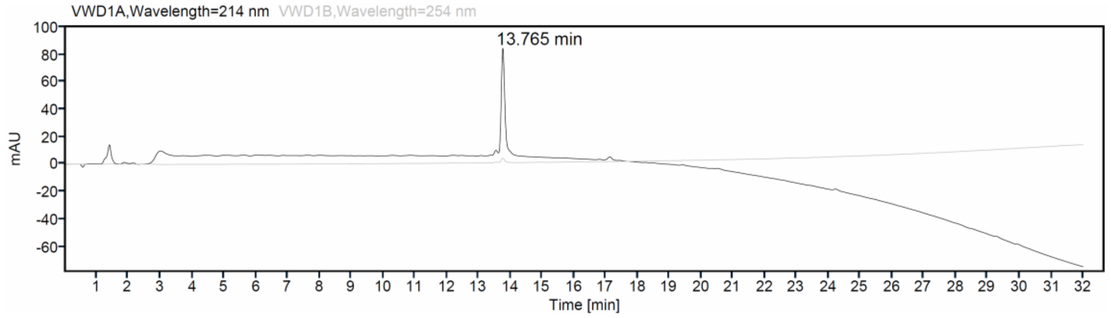

SI Figure 15. UHPLC profile of [T5A]GLP1 pure product. *R*t 13.765 min (Agilent Zorbax 300SB-C18 column, 5 μm, 2.1 × 150 mm, 5-95% MeCN over 30 min, *ca.* 3% MeCN/min), 95% purity based on Area Under Curve (AUC) at λ = 214 nm, accounting for all peaks between 4.0–29 min.

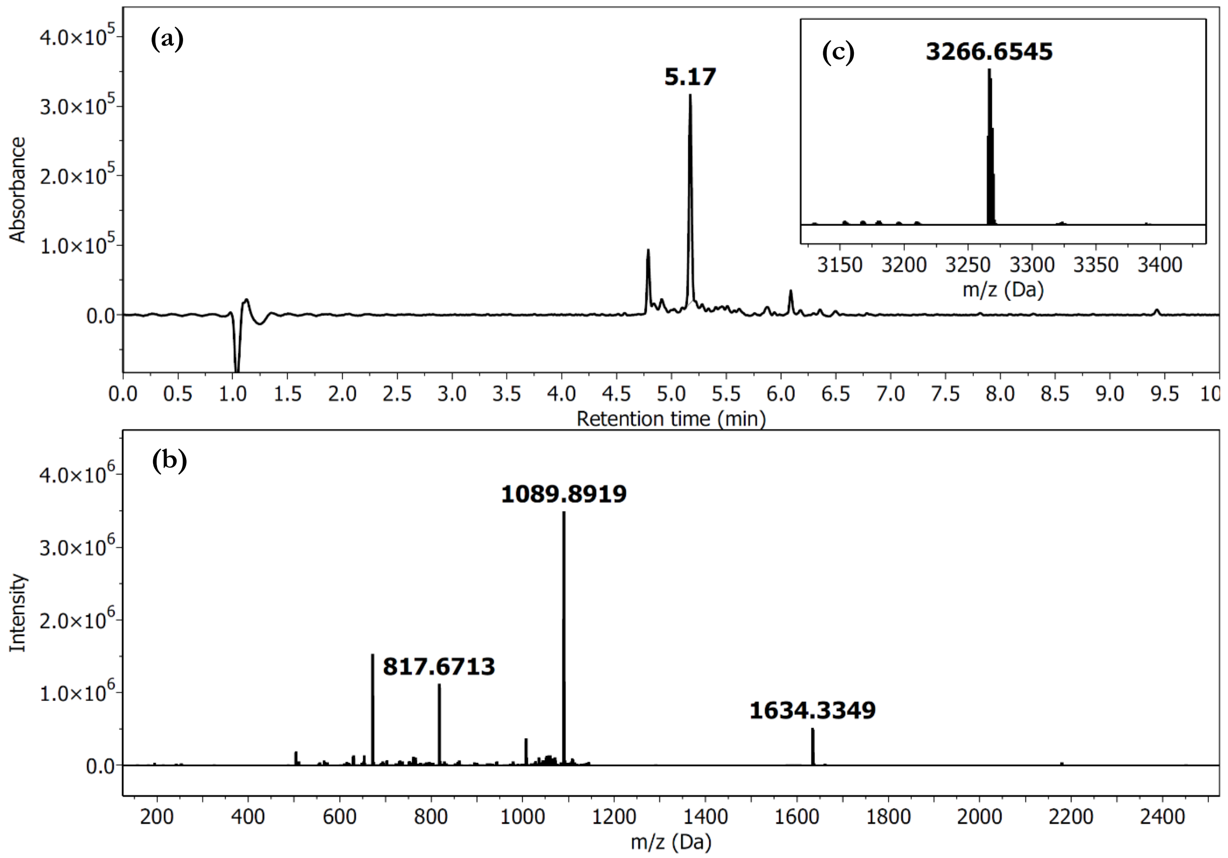

SI Figure 16. LCMS profile of crude peptide [T5A]GLP1. (**a**) Chromatogram (λ = 214 nm) of crude [T5A]GLP1; *R*t 5.172 min. (**b**) HRMS (ESI-TOF) spectrum. (**c**) Deconvoluted HRMS; monoisotopic mass (ESI+) calcd. for $C_{148}H_{224}N_{40}O_{44}$ 3265.6520, found 3265.6516.

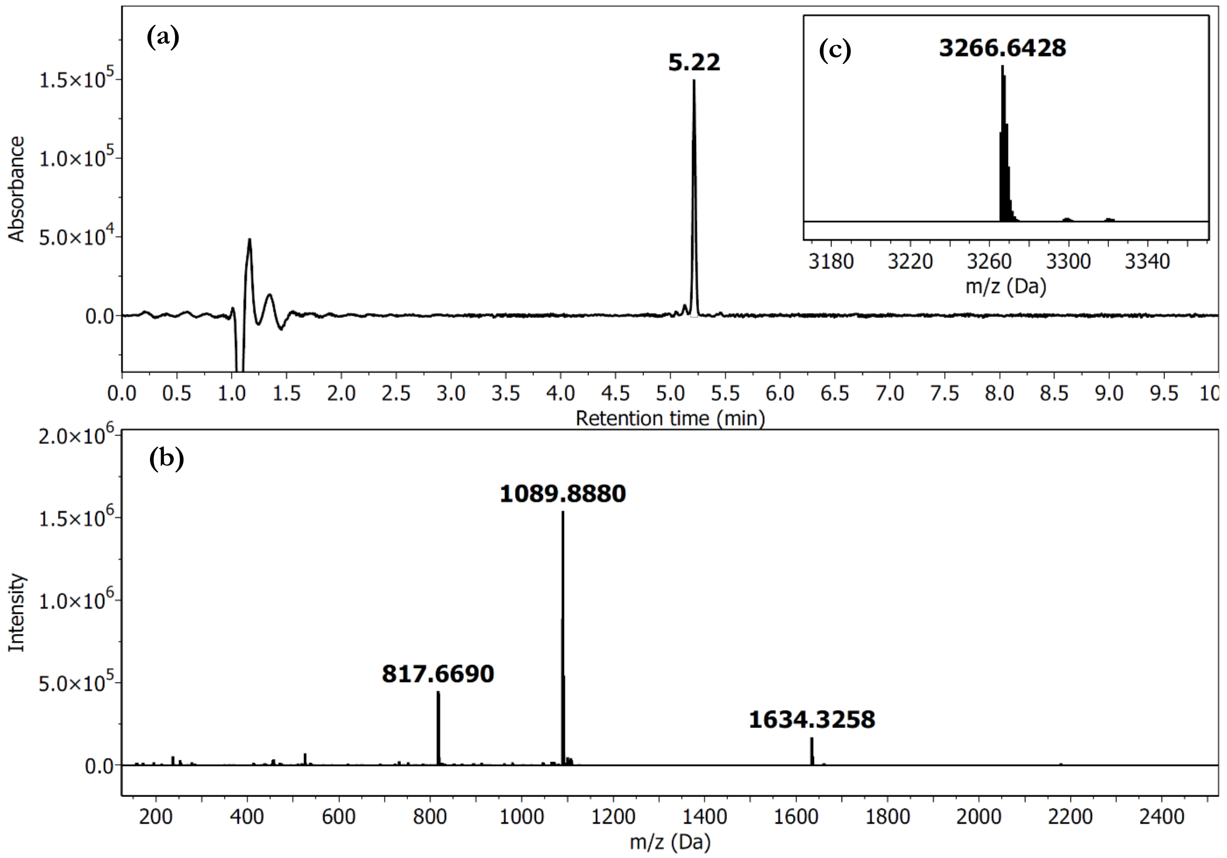

SI Figure 17. LCMS profile of pure peptide [T5A]GLP1. (**a**) Chromatogram (λ = 214 nm) of pure [T5A]GLP1; *R*t 5.215 min. (**b**) HRMS (ESI-TOF) spectrum. (**c**) Deconvoluted HRMS; monoisotopic mass (ESI+) calcd. for $C_{148}H_{224}N_{40}O_{44}$ 3265.6520, found 3265.6399.

Synthesis of Photo-GLP1

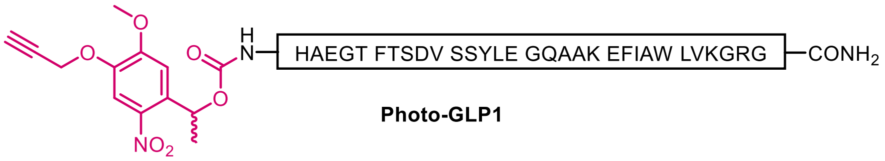

The peptide GLP-1 was first prepared via AFPS using Novabiochem® NovaPEG Rink Amide resin (0.20 mmol/g, 0.16 g, 32 μmol) using methods outlined in **General Procedure 2.1**. Total synthesis time to afford resin-bound GLP-1 (0.24 g final resin weight) was approximately 1.5 h. The peptidyl-resin (79 mg, approx. 10 μmol, 1.0 eq.) was swelled in DMF (2 mL) for 10 min, then drained. A solution of 1-(5-methoxy-2-nitro-4-(prop-2-yn-1-yloxy)phenyl)ethyl *N*-succinimidyl carbonate (**SI1**) (39 mg, 0.10 mmol, 10 eq.) and *i*Pr_2NEt (35 μL, 0.20 mmol, 20 eq.) in DMF (2 mL) was added to the pre-swelled peptidyl-resin, and the reaction was stirred at room temperature for 1 h. The peptidyl-resin was then drained, washed with DMF (3 × 2 mL) and CH_2Cl_2 (3 × 2 mL), then dried under reduced pressure to afford resin-bound Photo-GLP1 (68 mg final resin weight). Cleavage of the peptidyl-resin (32 mg) was carried out using **General Procedure 2.2** to afford the crude peptide (5.9 mg, 49% purity by UHPLC, monoisotopic mass calc. 3629.7427, found 3629.2054) **SI Figures 18** and **20**. The crude peptide (5.9 mg) was purified by semi-prep RP-HPLC using **General Procedure 2.3** with a gradient of 10–95% B over 170 min (*ca.* 0.5% B/min) on an Agilent Zorbax 300SB-C8 column at room temperature. Fractions were analyzed by LCMS and UHPLC, and fractions containing the correct m/z and high purity (>95% purity by UHPLC) were combined and lyophilized to afford the *title compound* (0.89 mg, >95% purity by UHPLC, 5% overall yield, monoisotopic mass calc. 3629.7427, found 3629.2373), **SI Figures 19** and **21**.

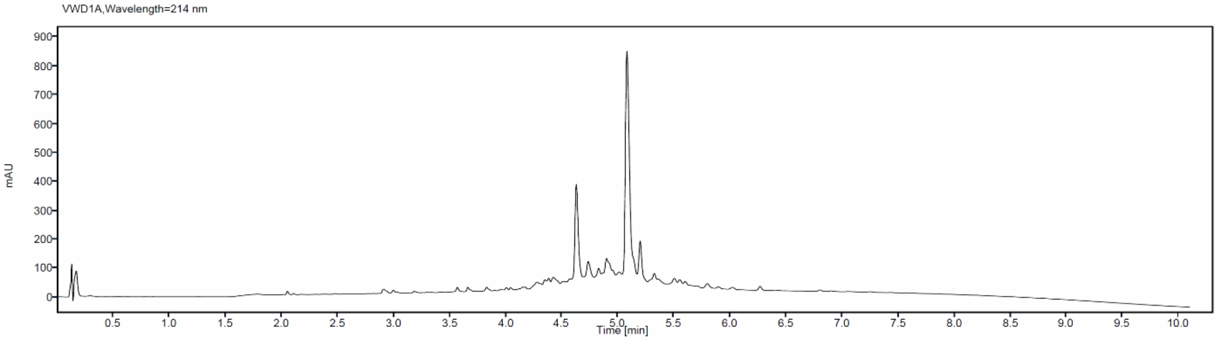

SI Figure 18. UHPLC profile of crude photo-GLP1; Rt 5.081 min (Agilent Zorbax Eclipse Plus C18 Rapid Resolution HD column, 1.8 μm, 2.1 × 50 mm, 5-95% MeCN over 9 min, *ca.* 10% MeCN/min), 49% purity based on Area Under Curve (AUC) at λ = 214 nm, accounting for all peaks between 2.0–9.5 min.

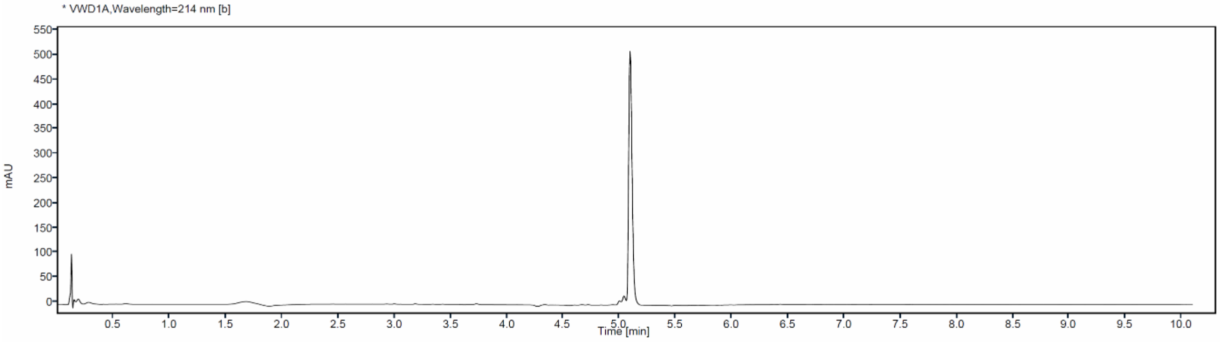

SI Figure 19. UHPLC profile of pure photo-GLP1; Rt 5.097 min (Agilent Zorbax Eclipse Plus C18 Rapid Resolution HD column, 1.8 μm, 2.1 × 50 mm, 5-95% MeCN over 9 min, *ca.* 10% MeCN/min), >95% purity based on Area Under Curve (AUC) at λ = 214 nm, accounting for all peaks between 2.0–9.5 min

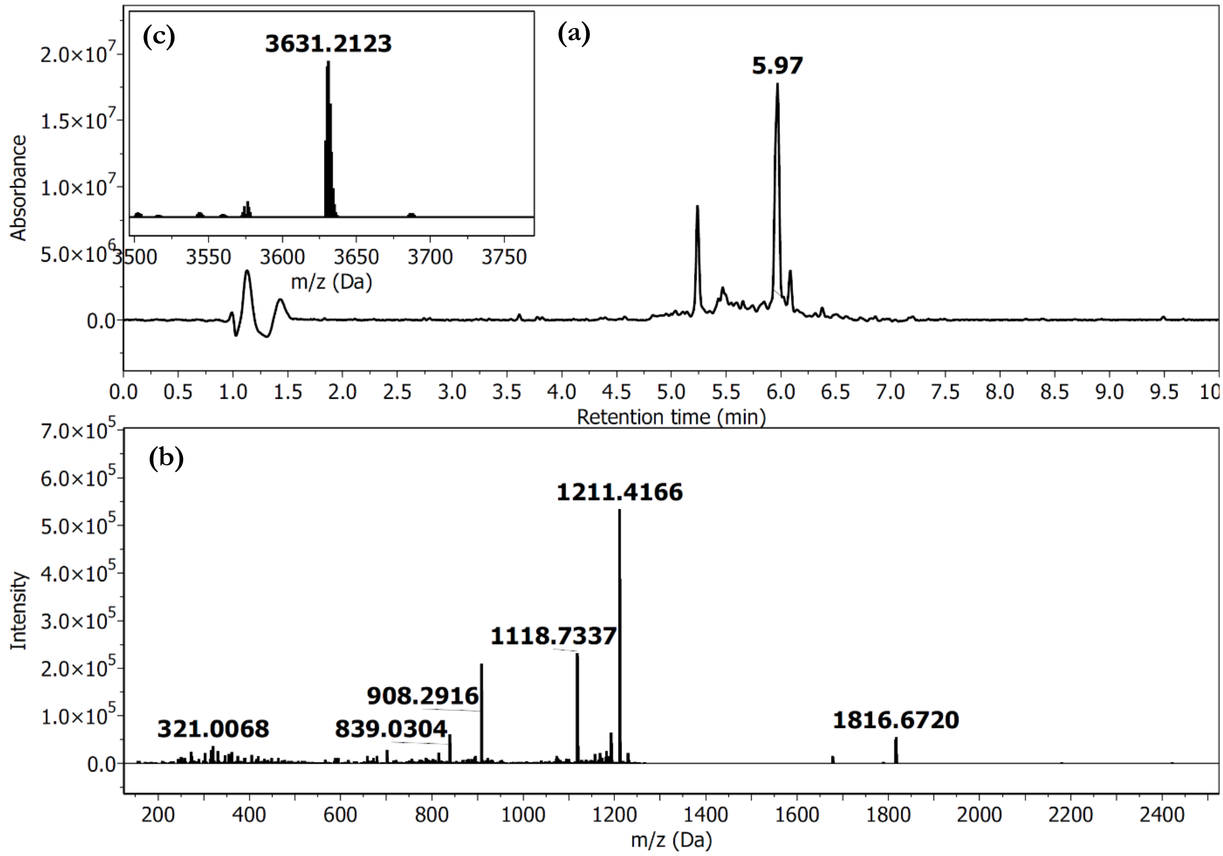

SI Figure 20. LCMS profile of crude photo-GLP1. (**a**) Chromatogram (λ = 214 nm) of crude photo-GLP1; *R*t 5.97 min. (**b**) HRMS (ESI-TOF) spectrum. (**c**) Deconvoluted HRMS; monoisotopic mass (ESI+) calcd. for $C_{164}H_{240}N_{42}O_{52}$ 3629.7427, found 3629. 2054.

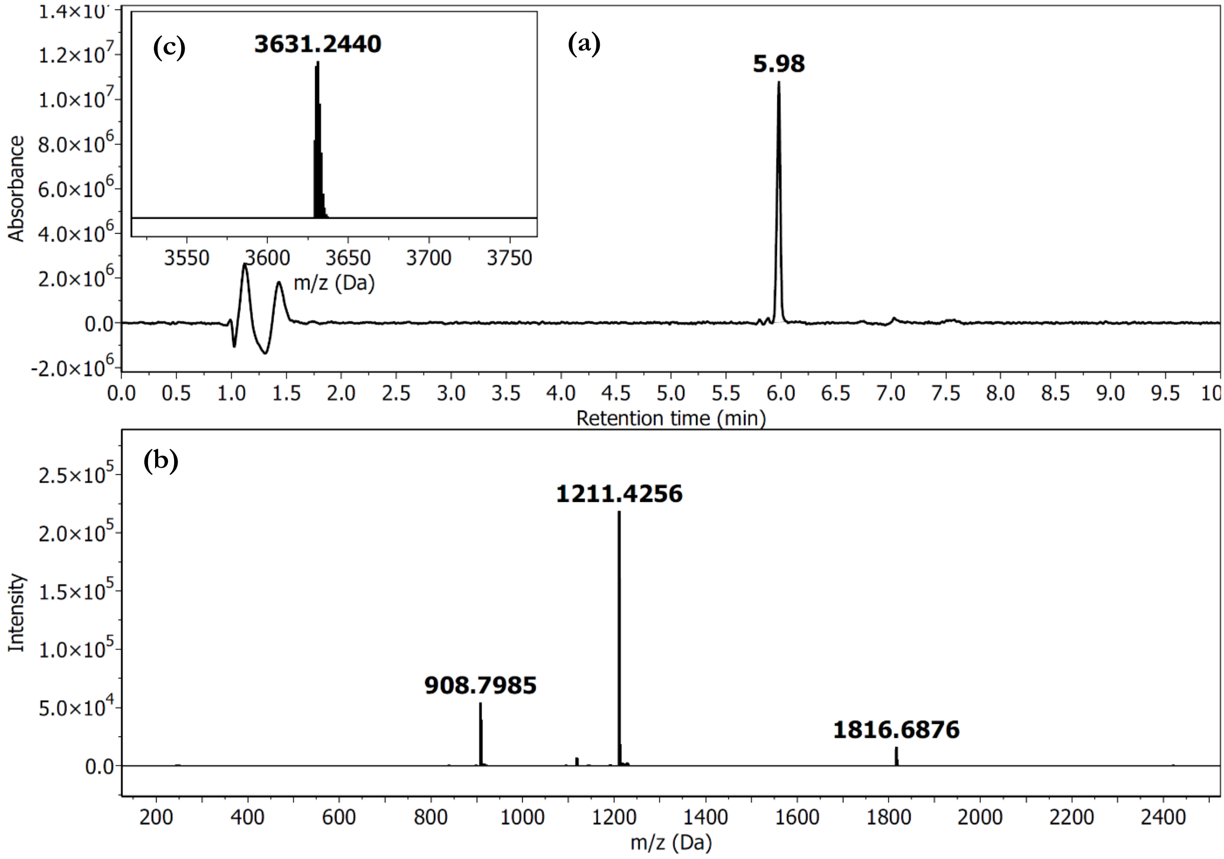

SI Figure 21. LCMS profile of pure photo-GLP1. (**a**) Chromatogram (λ = 214 nm) of pure photo-GLP1; *R*t 5.98 min. (**b**) HRMS (ESI-TOF) spectrum. (**c**) Deconvoluted HRMS; monoisotopic mass (ESI+) calcd. for $C_{164}H_{240}N_{42}O_{52}$ 3629.7427, found 3629.2373.

5 SI References

[1] N. Hartrampf, A. Saebi, M. Poskus, Z. P. Gates, A. J. Callahan, A. E. Cowfer, S. Hanna, S. Antilla, C. K. Schissel, A. J. Quartararo, X. Ye, A. J. Mijalis, M. D. Simon, A. Loas, S. Liu, C. Jessen, T. E. Nielsen, B. L. Pentelute, *Science* **2020**, *368*, 980–987.

[2] K. K.-S. Tso, K.-K. Leung, H.-W. Liu, K. K.-W. Lo, *Chem. Commun.* **2016**, *52*, 4557–4560.

[3] S. Kaneko, H. Nakayama, Y. Yoshino, D. Fushimi, K. Yamaguchi, Y. Horiike, J. Nakanishi, *Phys. Chem. Chem. Phys.* **2011**, *13*, 4051–4059.
